# Relationship between pre- and post-operative isokinetic strength after ACL reconstruction using hamstring autograft

**DOI:** 10.1186/s13102-020-00215-7

**Published:** 2020-10-30

**Authors:** J. Riesterer, M. Mauch, J. Paul, D. Gehring, R. Ritzmann, M. Wenning

**Affiliations:** 1 Rennbahnklinik, Muttenz, Basel, Switzerland; 2 Department of Sport and Sport Science, University of Freiburg, Freiburg, Germany; 3 Department of Orthopedic and Trauma Surgery, Medical Faculty, University Medical Center, Freiburg, Germany

**Keywords:** Isokinetic strength measurements, ACL reconstruction, Return to sport, Pre-habilitation

## Abstract

**Background:**

Anterior cruciate ligament (ACL) ruptures are of major concern in sports. As mostly young and active individuals are affected there is an emphasis on the rapid and safe return to sports (RTS). Strengthening the ventral and dorsal thigh muscles is a prerequisite for a successful RTS after ACL reconstruction (ACLR), as persistent muscle weakness may increase the incidence for secondary injuries and impair performance. Aiming to increase evidence on the importance of preoperative muscle strength and the coaching of patients, the purpose of this study is to compare thigh muscle strength pre- and post-operatively after ACLR.

**Methods:**

We performed a retrospective analysis of 80 patients with primary, isolated ACLR using a four-stranded hamstring autograft. We performed bilateral isokinetic concentric strength measurement (60°/s) before and six months after ACLR. Primary outcomes were the maximal knee extension and flexion torque, hamstrings-to-quadriceps ratio (H/Q ratio) and the corresponding limb symmetry indices (LSI). Pearson correlations were calculated for pre- and post-surgical values.

**Results:**

The operated as well as the unaffected leg increased maximal knee extension (+ 18% ± 7% *p* < 0.05; + 11% ± 5% p < 0.05) and flexion torque (+ 9% ± 5% p < 0.05, + 10% ± 6% p < 0.05) throughout the 6 months of rehabilitation. The H/Q ratio remained unaffected (− 2% ± 3% *p* = 0.93; − 4% ± 4% *p* = 0.27). LSI of knee extension strength increased significantly (6% ± 3% *p* < 0.05), while flexion strength remained unaffected (+ 2% ± 4% *p* = 0.27). Positive correlations underline the interrelationship between the strength pre- and post-surgery for the knee extension (r = 0.788 *p* < 0.05) and knee flexion strength (r = 0.637 p < 0.05) after ACLR.

**Conclusions:**

Preoperative leg extension and flexion strength normalized to body mass are strongly correlated to postoperative strength performance after ACLR. Therefore, pre-operative quadriceps and hamstring muscle strength deficits may have a significant negative impact on functional performance following ACLR. This emphasizes the need for intensive preoperative screening and subsequent treatment to achieve the best possible preoperative leg strength before ACLR.

**Trial registration:**

DRKS00020210.

## Background

Ruptures of the anterior cruciate ligament (ACL) occur with an incidence of 80 per 100,000 individuals each year [1]. ACL rupture causes knee instability, deficiencies in motor control and impaired arthrokinematics [2]. Primary injury and chronic instability are often accompanied by a progressive loss of muscular strength [3, 4] which may cause cartilage and meniscal damage favoring the development of osteoarthritis [5]. Mechanical, proprioceptive and efferent neuromuscular impairments contribute to muscular deficits [6]. Before a successful return to sport (RTS) can be achieved, a recovery of balanced bilateral and ipsilateral strength and functional performance is essential [3].

While surgical ACL reconstruction (ACLR) is aiming to restore kinematics, joint function and stability, postoperative rehabilitation is designed to restore active stabilization of the knee and neuromuscular function [7, 8]. The optimal integration of active and passive stabilizers will allow for a safe and successful RTS [2]. Apart from reduced physical performance, factors such as arthrogenic muscle inhibition (AMI) [9] and impaired proprioception of the joint contribute to the development of post-operative muscle weakness and atrophy [10]. Immediately after injury, AMI has a protective function, whereas after ACLR, AMI can be interpreted as a limiting factor of post-operative strength recovery [6, 11] associated with persisting insufficiency of the quadriceps and hamstring musculature [12].

A recent study suggested that preoperative quadriceps strength is associated with improved postoperative strength and knee function [13]. Systematic preparatory interventions and adapted training protocols prior to surgery, so-called pre-rehabilitation, may prove to be a valuable and valid therapeutic intervention. While numerous studies provide scientific evidence for the choice of exercises, progression schemes and efficiency [14–16] for rehabilitation algorithms after ACLR, the evidence on benefits of improving preoperative strength status is still scarce. Although fundamental data obtained in healthy populations made it apparent that the force generating capacity prior to (partial-) immobilization predicts the post-interventional torque [17], this interrelation has not been established clinically in orthopedic patients suffering from an ACL rupture, yet. Previous findings [18] showed that a pre-operative deficit of more than 20% leads to a persisting reduction of strength up to two years after surgery. Likewise, Ueda, Matsushita [19] recommend a pre-operative quadriceps index of a minimum of 70% in order to achieve an LSI of at least 85% six months after ACLR. We therefore conclude that bringing pre-operative factors to the fore will provide additional knowledge and strategic advance in clinical treatment and training after orthopedic traumata. This may underline the importance of immediate physiotherapeutic treatment before surgery in an effort to improve post-operative recovery and subsequent outcomes [14].

Thus, the objective of this study was to investigate the strength of the relationship between pre- and post-operative isokinetic strength of knee extensors and flexors, the corresponding limb symmetry index (LSI) and Hamstrings-to-quadriceps ratio (H/Q-ratio) for both legs in a large and homogenous cohort of athletes. The parameters have been chosen with reference to Schmitt, Paterno [20] and Coombs and Garbutt [21], who justified their significance for the RTS. Postoperative data was assessed approximately six months after surgery as it has been previously defined as the earliest time point after ACLR at which mobility, strength and neuromuscular control can be sufficiently restored to consider returning to sport-specific rehabilitation [22].

We hypothesized that the pre-operative maximum strength of thigh muscles as well as the pre-operative LSI correlate with the maximum strength and LSI six months after ACL reconstruction. As the strength of the ventral and dorsal thigh muscles was expected to adapt proportionally to partial unloading after ACL reconstruction and subsequent rehabilitation, the H/Q-ratio was hypothesized to remain constant.

## Methods

### Experimental approach to the study

**Fig. 1:**
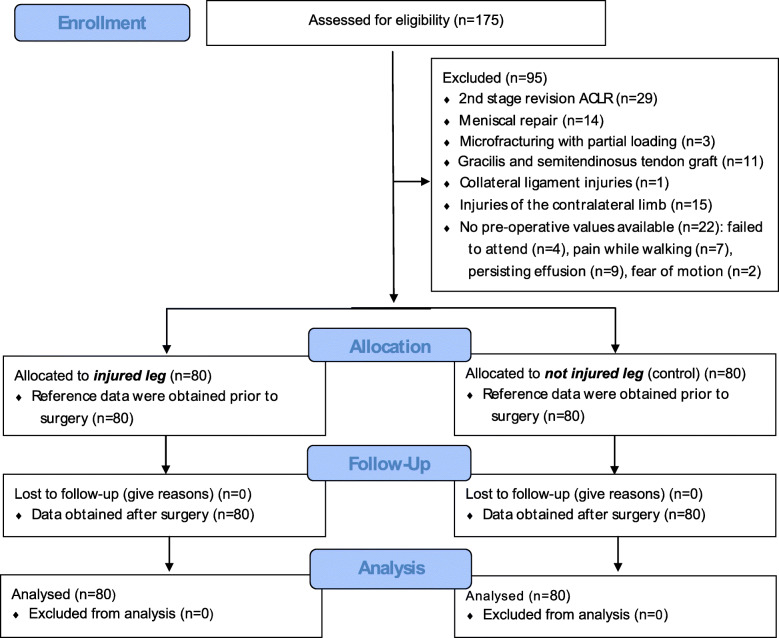
Flow chart diagram – description of the study population, inclusion and exclusion criteria

We performed a retrospective analysis in a repeated measurement design with a human sample of volunteers who experienced a primary ACL rupture. Isokinetic strength from knee extensor and flexor measurements were taken from clinical routine procedures pre-operatively and 6 months (26 ± 1 weeks) after ACLR to answer the research questions (Fig. 1). Data assessment was part of the clinical procedure which is running routinely.

### Subjects

The random sample consisted of *n* = 80 patients (54 male, 26 female) with an average age of 29 ± 9 years and an age range from 14 to 51 years. The sample size was estimated by means of a power analysis based on experimental evidence obtained from Eitzen et al. [18] and Ueda et al. (f = 0.90; alpha = 0.05; power = 0.90) [19]. The inclusion criterion was the reconstruction of ACL using a quadrupled, single-bundle ipsilateral semitendinosus autograft. Exclusion criteria were concomitant surgical interventions such as meniscal repair, collateral ligament reconstruction, cartilage procedures or osteotomies. Revision cases, patellar-tendon or allograft transplants as well as injuries of the contralateral limb or concurrent musculoskeletal problems were also excluded from this analysis. In cases of persisting, relevant effusion, pain while walking or restricted ranges of motion no isokinetic strength measurement was performed and thus patients were excluded (Fig. 1). In 58% of the subjects (*n* = 46) the right knee joint was injured. The average time between the injury and the surgery was 28 ± 61 weeks. Participant enrollment and eligibility criteria aiming to homogenize the sample (i.e. surgical treatment, graft side, rehabilitation protocol) and achieve accurate and clinically meaningful results with regard to RTS are described in Fig. 1. All subjects were informed of the benefits and risks of the study prior to giving written informed consent to the experimental procedure, which was approved by the local ethics committee and conducted in accordance with the latest version of the Declaration of Helsinki.

### Surgical treatment

The surgical technique was standardized for all subjects in this study. All participants received an arthroscopically-assisted ACLR using a quadrupled, single-bundle ipsilateral semitendinosus autograft. During surgery a tourniquet was placed around the proximal thigh. Femoral tunnel drilling was performed through the anteromedial portal with an extracortical femoral fixation and an aperture tibial fixation using a bioresorbable interference screw and additional extracortical fixation.

### Rehabilitation algorithm: protocol and diagnostics

Standardized post-surgical treatment was achieved by a homogenous controlled time-and evidence-based rehabilitation protocol accompanied by time-normalized diagnostics as a clinical routine procedure. The algorithm was identical and obligate for all patients and executed with both the injured and not injured leg with reference to Adams, Logerstedt [14], Fukuda, Fingerhut [15] and Kruse, Gray [16] as illustrated in Table 1. The criterion-based rehabilitation scheme of the current study - adopted from Keller, Kurz [23] – contained a clustering into four phases with a progressive therapy algorithm respecting the vulnerability of the bony and ligamentous tissue. Exercises ranged from monoarticular passive treatment to multiarticular active strengthening up to functional tasks with high load. Patients and therapists were given written information about the procedure, exercises and progress criteria by the surgeon. The implementation of and adherence to the rehabilitation scheme were controlled for by the physiotherapists.

**Table 1:** Time and criterion-based rehabilitation scheme for the first six months after ACL reconstruction [14–16] divided into 5 phases (first column). Objectives (middle columns) and interventions (right column) contain the therapy and diagnostics. Note that the retrospective data analysis are based on the diagnostical measures frames in black

Phase	Objectives	Physiotherapeutic and diagnostic scheme
Prior to surgery	- Conservative preservation of skeletal muscle mass and function (immediately after the diagnosed injury)	- Alleviation of pain - swelling measures *-* Detonisation and lymph measures - Proprioceptive training - Quadriceps muscle strength training
	- *Biomechanical diagnostics to assess muscle function and strength prior to surgery*	- *Isokinetic force measurement of thigh extensors and flexors* *- Concentric isokinetic protocol: 60°/s (extension) – 60°/s (flexion)*
Phase 1 (week 1–2)	- Angular mobility in flexion and extension (90/0/0) - Isometric quadriceps activation - Activities of daily living	- Alleviation of pain and stiffness - Passive and active knee mobilization - Patella mobilization - Isometric quadriceps activation - Gait training (with walking sticks) - Proprioceptive training (bipedal) - Bicycle ergometer *-* Neuromuscular stimulation with Compex®
Phase 2 (week 3–6)	*-* Reduction of pain and detonisation - Angular mobility in flexion and extension (> 110/0/0) - Progressive improvement in muscle-coordination *-* Normalization of gait pattern *-* Stair climbing	- Passive and active knee mobilization - Gait training (without walking sticks) - Proprioceptive training one-legged - Coordination in closed kinematic chain (Squat, Squat lunges) - Hip and core stability training - Bicycle ergometer - Neuromuscular stimulation with Compex®
Phase 3 (week 7–12)	- Symmetrical knee mobility and active range of motion - Running, cycling and crawl-swimming	- Strength development (maximal strength) in open and closed kinematic chain - Increasing proprioceptive training - Running and jumping “ABC” with stable leg axis *-* Jogging (symptom-free running and jumping is a prerequisite)
Phase 4 (week 13–26)	- stretch shortening cycle without pain - Jogging outdoors	- Progressive running and jumping “ABC” with stable leg axis - Continuation of maximal strength development - Explosive strength *-* Stretch shortening exercises including ballistic jumps i.e. hops and drop jumps, squat and countermovement jumps
	- *Biomechanical diagnostics approx. 6 months after surgery diagnostics to assess muscle function and strength prior to surgery to evaluate target-orientated therapy*	- *Isokinetic force measurement of thigh extensors and flexors* *- Concentric isokinetic protocol: 60°/s (extension) – 60°/s (flexion)*

For diagnostics to classify the patients we have evaluated the isokinetic force of the thigh extensors and flexors first ±1 week before the operation and second 6 months (±2 weeks) after the operation.

#### Isokinetic strength measurement

Knee extensor and flexor strength was assessed using an isokinetic dynamometer (Humac®/NormTM Testing & Rehabilitation System, Computer Sports Medicine, Inc. (CSMi, Stoughton, Massachusetts, US) according to Li, Wu [24]. The dynamometer was calibrated prior to testing sessions.

**Fig. 2:**
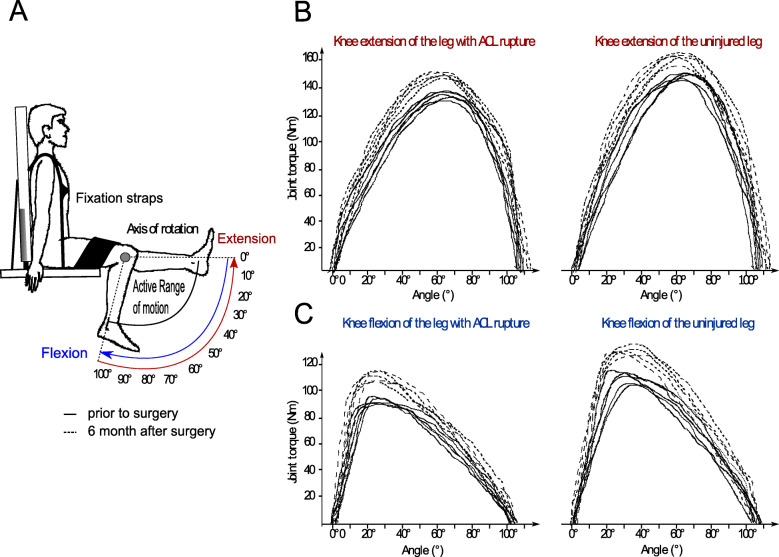
Isokinetic strength measurements of the knee extensors and flexors. **a** Body position, axis of rotation and the amplitude of the active ROM for both movement directions established in a concentric test setting. **b** Knee extension and **c** knee flexion joint torque with reference to the knee angle displayed for the ACL injured and not injured leg pre (−---) and post (− − - -) surgery of one subject of the random sample

Each subject was placed in an upright sitting position, the trunk at 100° leaning against the back rest of the testing table, fixed by straps across the chest and a horizontal pad over the middle third and proximal half of the distal third of the thighs [25]. The knee joint axis was aligned with the mechanical axis of the dynamometer [24]. The shin pad was placed just superior to the medial malleolus (Fig. 2).

Prior to each test sequence independent of the time of day, each subject performed the same and standardized 10 min warm up protocol on a cycling ergometer followed by three submaximal repetitions to familiarize with the testing procedure. For data assessment we use the protocol of Li, Wu [24] of concentric-concentric contractions a 60°/s angular speed, in the full individual range of motion (ROM) due to its high test-retest reliability [26]. Two sets of five repetitions with maximum effort were executed (Fig. 2). Each trial was initiated with the unaffected limb. Between sets, patients had at least rest for 1 min [26].

Outcome parameters were: maximal knee extension and flexion torque normalized to body mass (Nm/kg) [27], the H/Q-ratio [14] and the limb symmetry index (affected limb/unaffected limb*100) for the knee extensors and flexors [19, 28]. For data assessment, the better of the two sets was selected.

### Statistical analysis

Statistical analyses were executed using SPSS 23 (SPSS Inc., Chicago, Illinois). The effects of ACLR on the variables knee extensor and flexor peak torque, the LSI and the H/Q-ratio were evaluated using a two-factor analysis of variance (ANOVA); time (pre vs. post) and affection (affected vs. unaffected leg). Prior, the normality of the data was evaluated using the Kolmogorov–Smirnov test, which indicated that the data followed a normal distribution. The level of significance was set to > 0.05.

Bivariate, two-tailed Pearson correlation analyses were conducted to determine the strength of the linear relationship between the dependent variables obtained before and after surgical treatment. Correlation strength was interpreted according to Cohen as followed: < 0.3 weak correlation, 0.3–0.5 moderate correlation, > 0.50 strong correlation [29]. Simple linear regression models were used to estimate the predictably of strength values assessed after surgical treatment as the dependent variable as a function of values obtained prior to ACLR as the independent variable.

Values are presented as mean values ± standard deviations (M ± SD).

## Results

Grand means of the maximal knee extension and flexion torque normalized to body mass, the LSI and the H/Q ratio for the affected and unaffected leg are presented in Table 2. The ANOVA revealed no significant time x affection interaction effects.

**Table 2:** Maximum strength is displayed as the knee extension and flexion torque normalized to body mass [Nm/kg] (Mean ± SD), Limb Symmetry Index and the H/Q ratio [%] pre- and post- surgical reconstruction of the ACL

	Limb	Pre-operative (M ± SD)	6 months post-operative (M ± SD)	Change pre to post (%)	rmANOVA (p, F)
Knee extension torque [Nm/kg] (M ± SD)	affected	1.3 ± 0.5	1.6 ± 0.5	+ 18 ± 7	*P* < 0.05, F = 8.3
	unaffected	1.7 ± 0.5	1.9 ± 0.4	+ 11 ± 5	P < 0.05, F = 4.7
Limb Symmetry Index		79 ± 23	84 ± 16	+ 6 ± 4	*P* < 0.05, F = 3.0
Knee flexion torque [Nm/kg] (M ± SD)	affected	1.0 ± 0.3	1.1 ± 0.3	+ 9 ± 5	P < 0.05, F = 3.0
	unaffected	1.2 ± 0.31	1.3 ± 0.3	+ 10 ± 6	P < 0.05, F = 5.2
Limb symmetry index		82 ± 19	84 ± 13	+ 2 ± 4	*P* = 0.36, F = 0.6
H/Q-ratio (%)	affected	81 ± 31	79 ± 20	− 2 ± 5	*P* = 0.93, F = 0.2
	unaffected	74 ± 13	71 ± 13	− 4 ± 4	*P* = 0.27, F = 0.9

**Fig. 3:**
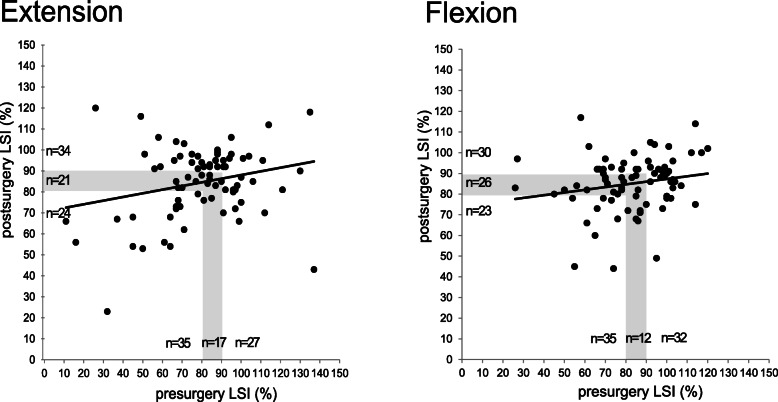
Descriptive distribution of pre- and postoperative LSI

Time effects reached statistical significance for the peak torques normalized to body weight for the knee extension and flexion of the affected and unaffected leg indicating an increase within 6 months of rehabilitation process independent of the injury side and contra-laterality. The thigh muscles of the affected leg after 6 months had not reached equivalent joint torques realized by the unaffected leg prior to surgery. The H/Q ratio showed no significant changes in both legs 6 months after ACL reconstruction comparable to the values obtained prior to surgery (Fig. 3).

The LSI between the pre- and post-operative values for the knee extension increased significantly, while the LSI for the knee flexion remained comparable to the pre-operative level. Sub-analysis showed that less than 50% of the patients (knee extension *n* = 34 and flexion *n* = 30) had achieved an LSI > 90% after 6 months (Fig. 3). Of the *n* = 27 patients that had a pre-surgery LSI > 90% in knee extension strength, 44% (*n* = 12) had recovered an LSI of > 90% after 6 months while 33% (*n* = 9) achieved an LSI between 80 and 90% and 22% (*n* = 6) remained with an LSI below 80%. On the other end, of the initial *n* = 35 patients with a pre-operative LSI < 80% in extension strength, there were 37% (*n* = 13) that achieved an LSI > 90% post-operatively, while n = 6 had an LSI between 80 and 90% and 43% (*n* = 16) still remained with an LSI < 80%.

**Fig. 4:**
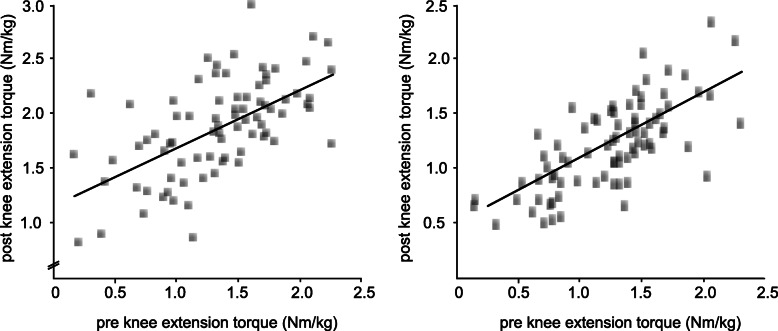
The graph illustrates the scatter plot, regression line and regression equation for the relationship between the dependent variables maximal isokinetic knee extension (left) and flexion torques (right) normalized to body mass of the injured leg obtained before (abscissa) and after (ordinate) ACL reconstruction. R^2^ linear is the coefficient of determination; the fitted regression line is described by the equitation y

Bivariate correlation coefficients R and *p*-values for the dependent variables are displayed in Table 3, regressions are illustrated in Fig. 4. Positive correlations were detected between pre- and post-operative knee extension and flexion torques for the injured and surgically treated leg with an ACLR. Simple linear regressions revealed a significant positive association between pre- and post-surgical strength for knee extension (*p* < 0.05, F = 14.04) and flexion (p < 0.05, F = 22.82). The LSI showed no significant correlations or correlations of minor statistical significance with correlation coefficients considered as weak.

**Table 3:** Correlation coefficients for bivariate Pearson correlations between the two different time points (pre-operative and 6 months postoperative). *p < 0.05, bold values represent strong correlations

			Post-operative			
			Extension strength		Flexion strength	
			affected leg	LSI	affected leg	LSI
pre-operative	**Extension strength**	Affected leg	0.79*	0.29	0.63*	0.16
		LSI	0.33*	0.18	0.26*	0.06
	**Flexion strength**	Affected leg	0.5*	0.09	0.64*	0.11
		LSI	0.36*	0.15	0.39*	0.22

## Discussion

This study provides evidence for the interrelationship of isokinetic strength performance before and 6 months after ACLR. The findings support the use of pre-rehabilitative interventions for the postoperative recovery of thigh muscle strength. Results are based on a longitudinal assessment in a homogenous cohort of athletes undergoing ACLR which reiterates the importance of detailed analysis of strength performance, limb symmetry (LSI) and ipsilateral strength balance.

The present findings confirm the presumption that preoperative extension and flexion strength normalized to body mass are strongly correlated to postoperative strength performance. Thus, these results provide strong evidence that patients benefit from improved pre-operative strength performance, especially in the affected leg [13], as these values seem to be of major impact when considering progressive rehabilitation at least during the six months following ACL reconstruction [18]. Factors underlying this time-dependent relationship are likely attributed to the persistency of skeletal muscle structure and neuronal factors [30, 31]. These factors seem to affect the knee extensors and flexors equally as indicated by the consistency in H/Q-ratios. Thereby, muscles which experienced hypertrophic stimuli throughout exercising and improved their cross sectional area are less effected by periods of partial weight bearing or immobilization [31, 32] as it occurs after surgery [33]. Although we expect our patients to experience atrophy during the resting period and phases of low impact sports after the surgery, these findings draw attention to the pre-surgical period and the potential benefits of utilizing this period for pre-habilitation [18, 19]. Postoperatively, there are numerous factors that can influence a successful rehabilitation and RTS, among which recovery of symmetric strength performance is currently postulated to be the most effective factor, that has been established [34]. The unequal increase in strength between the legs established by the LSI, that was observed in our study, may partially be attributed to the low pre-operative performance of the quadriceps muscle of the affected leg. Although its origin is still unknown, it has been frequently postulated that pre-operative deficits could originate from mechanical, psychological or neural factors like arthrogenic muscle inhibition [35–37]. Regardless of the cause, our results as well as the findings in the literature suggest, that patients benefit from pre-surgical improvement of muscular activation. The beneficial effect of pre-habilitation may be best imagined as the recovery of physiological neuromechanics as early as possible, in an effort to prepare for the negative impact (i.e. atrophy) expectable from the surgical intervention. With the intention to prepare physically before the surgery through physiotherapy and systematic training interventions to trigger hypertrophy and improve coordinative skills of the muscles encompassing and stabilizing the knee joint, a good patient outcome can be expected. Beside the discussion about structures and mechanisms underlying this interrelationship, the outcomes emphasize the potential for preoperative treatment to achieve the best possible preoperative strength before going into surgery.

Surprisingly, there was only a moderate correlation between the LSI pre- and post-operatively for both flexion and extension in the athlete population. Side effects arising from the study population [2, 5], persistent arthrogenic muscle inhibition [35–37] or the period of half a year between surgery and postoperative assessment may have determined this outcome. However, another study has shown that a deficit of 20% of the LSI before surgery is associated with persisting deficits for a duration of up to two years postoperatively [18]. Furthermore, it has been stated that a pre-operative LSI in extension of at least 70% is recommended to reach an index of 85% six months after ACLR [19]. The presented data in this study show that a preoperative LSI > 80% was observed in 56% of the participants for extension and flexion strength. While limb symmetry has been an established parameter for assessing patients’ readiness for RTS, the current results referring to the LSI coupled with recent findings [4] question the significance of this parameter for RTS testing: Factors like leg dominance or primary sport, are contributing to pre-existing side-to-side differences even in healthy athletes. Generally, the achievable increment of strength during rehabilitation also depends on factors like joint condition (swelling, pain), general fitness level and psychological aspects including motivation and anxiety [11]. Yet, many of these parameters that may potentially be influencing LSI, have been poorly reported and defined.

Postoperatively, an LSI in strength > 90% is defined as a major RTS criterion [34]. In our study, 43% (extension) and 38% (flexion) of all patients reached an LSI > 90% in strength 6 months postoperatively. One other study reports that a Quadriceps index > 90% was achieved by 72.6% of the patients 6 months post-operatively, and by 78.2% 12 months post-operatively [38]. In the study of Palmieri-Smith and Lepley [39] only 20% of patients were able to achieve 90% quadriceps strength symmetry between limbs with an average LSI in knee extension strength of 71 ± 22%. Thus, in accordance with other recent publications, our findings underline that 6 months postoperatively the majority of patients is not yet adequately recovered for a RTS [34].

In general, even though LSI increases and shows a significant moderate correlation to preoperative values, high intraindividual variability needs to be respected. This is underlined by the sub-analysis showing that patients starting off with an LSI > 90% may still end up with an LSI < 80% postoperatively. On the other hand, patients with an initial LSI < 80% may go on to show the potential of recovering up to an LSI > 90%. Aiming to provide further evidence on the individual and perioperative factors contributing to postoperative limb symmetry a larger cohort and multivariate analysis is necessary. The multitude of an individual’s conditions including leg dominance, target sportive activity, contributing risk factors like dynamic valgus, tibial slope and insufficient landing strategies needs to be respected to attain a complete analysis.

### Limitations and strengths

When considering the limitations of the study, two aspects are of substantial importance: the first deals with the study design and the second with the sample. The study was not designed to investigate a causal relationship, nor specific adaptations during the rehabilitation in the contra-lateral limb. Furthermore, leg dominance and sport specific education may have had a significant impact. Further, the sample of 80 patients has been selected randomly in a given time interval. To homogenize the study population in regard to the main hypothesis and aim, we excluded patients who had received additional surgical procedures on co-morbidities. Therefore, the study outcomes are valid for patients with an ACL reconstruction without serious side injuries, but most probably not transferrable to other patient groups with multiple pathologies and co-injuries. The latter can also be considered a strength of the study, because it forms a very homogenous cohort and allows for a special focus on the research question. Further strengths of the article include its large sample size and the standardized surgical and rehabilitative procedures.

## Conclusions

This study of isokinetic thigh muscle strength of 80 patients before and 6 months after ACL reconstruction shows that preoperative thigh muscle strength correlates strongly with postoperative muscle strength. Thus, pre-operative quadriceps and hamstring muscle strength deficits appear to have significant negative functional consequences postoperatively. This underlines that prehabilitation has the potential to improve postoperative recovery of strength by reducing perioperative activation failure. However, there are several factors that can affect the LSI, such as leg dominance or an asymmetric sport like football, handball or athletics, where one leg is more relied upon and promoted than the other. This leg dominance in asymmetric sports is also taken up and further developed in sports-specific rehabilitation. This issue shows a clear bias to the LSI. In that case the LSI could show an inhomogeneous quotient which may be pathologically unimportant. The recovery of an adequate LSI has also a high interindividual variability and the factors contributing to the recovery of LSI before RTS will require further investigation.

## Data Availability

The datasets used and/or analyzed during the current study are available from the corresponding author on reasonable request.

## Abbreviations

ACL: Anterior cruciate ligament
ACLR: Anterior cruciate ligament reconstruction
H-Q-Ratio: Hamstrings-to-quadriceps ratio
LSI: Limb symmetry index
M: Mean
ROM: Range of motion
RTS: Return to sport
SD: Standard deviation
